# Supplementary material for: Involvement of 2-deoxyglucose-6-phosphate phosphatases in facilitating resilience against ionic and osmotic stress in Saccharomyces cerevisiae
Source: Microbiol Spectr. 2024 Jul 2;12(8):e00136-24. doi: 10.1128/spectrum.00136-24 (PMC11302306; doi:10.1128/spectrum.00136-24)

Involvement of 2-deoxyglucose-6-phosphate phosphatases in facilitating resilience against ionic and osmotic stress in *Saccharomyces cerevisiae*

Chinmayee Awasthy[1,#] ,Zeinab Hefny[1,#] ,Wouter Van Genechten[1], Uwe Himmelreich[2] and Patrick Van Dijck[1,*]

[1]Laboratory of Molecular Cell Biology, Institute of Botany and Microbiology, Kasteelpark Arenberg 31, B-3001 Leuven, Belgium

[2]Biomedical MRI/MoSAIC, Department of imaging and pathology, KU Leuven, Belgium

SUPPLEMENTARY FIGURES

Supplementary FIG S1. Overexpression of the *DOG* genes suppress the growth sensitive phenotype of the *gpp1Δ gpp2Δ* mutant. The liquid growth assays were done in medium containing 0.4 M NaCl (panel A) and, 0.6 M NaCl (panel B), or 12% sorbitol (panel C), 15% sorbitol (panel D) and, 18% sorbitol (panel E), for 72h as described in materials and methods. Growth (spot) assays was done on agar plates containing 0. 2 M, 0.6 M and, 0.8M NaCl (panel F) as well as 15% and 18% sorbitol (panel G), as described in materials and methods. 10-fold dilutions were spotted and visualized after 72h of growth at 30 °C. The data points are average of two independent experiments, each comprising three biological repeats. Each biological repeat is represented by the average of three technical repeats. Data are shown as average at each time point. The standard error of mean (SEM) is not shown as there was very little varition.

A

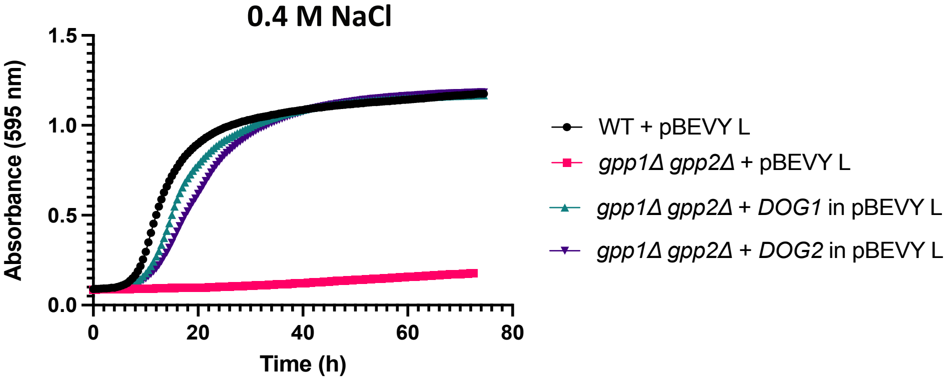

B

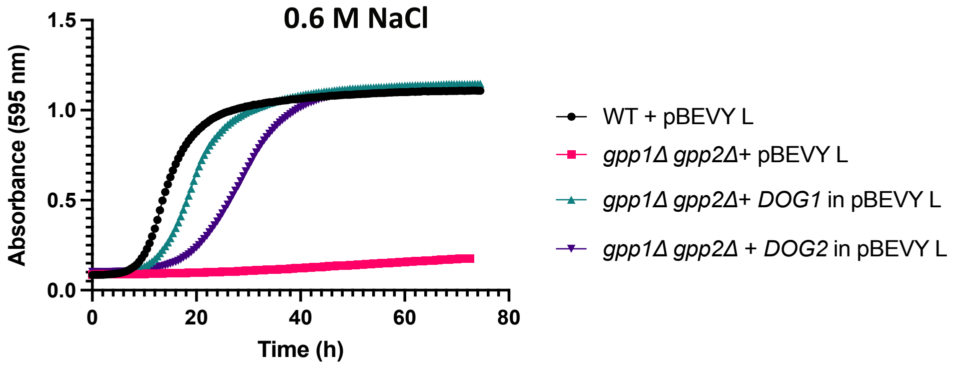

C

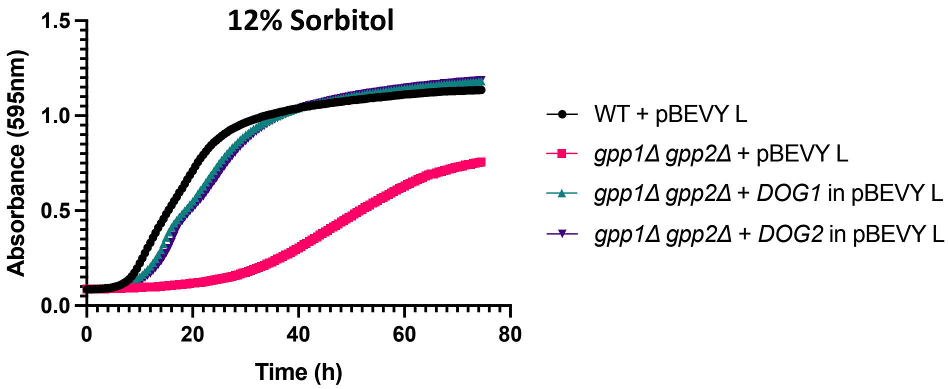

D

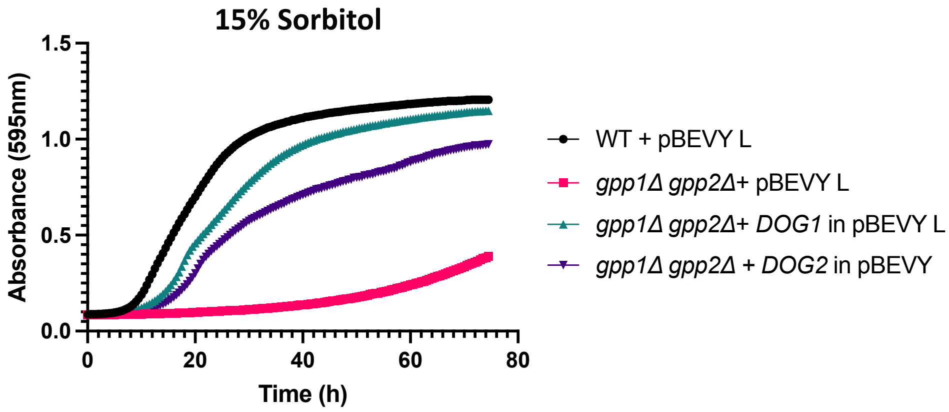

E

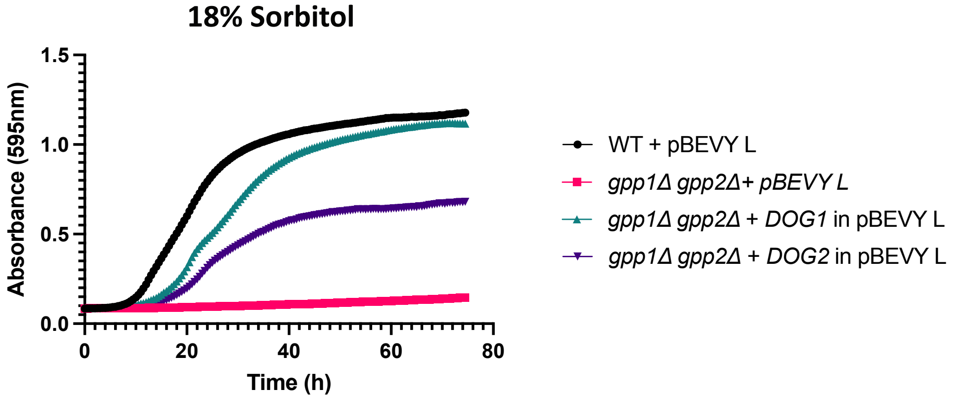

F

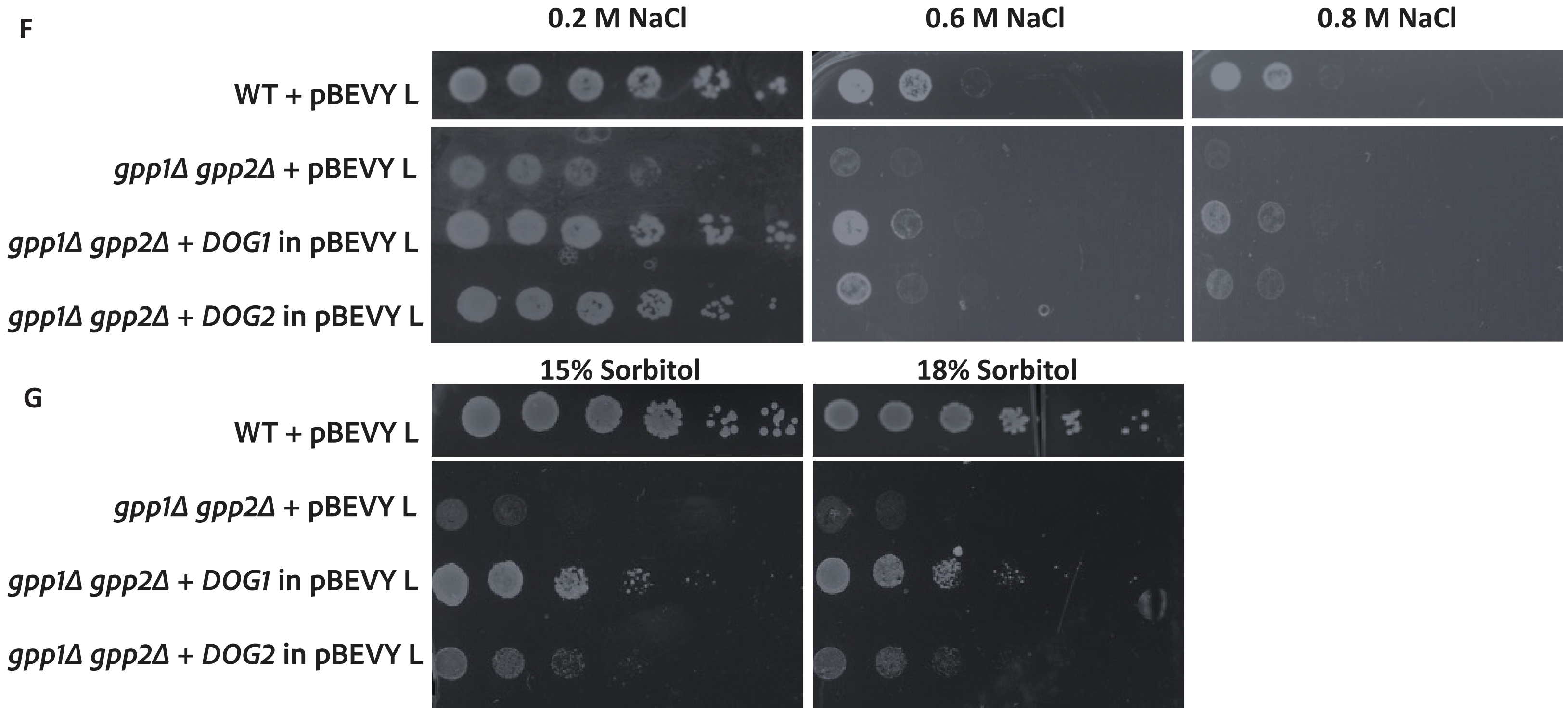

Supplementary FIG S2. *gpd1Δ gpd2Δ* mutant cease to grow under stress despite the overexpression of the *DOG* genes. The liquid growth assays were done in medium containing 0.4 M NaCl (panel A) and, 15% sorbitol (panel B), for 72h as described in materials and methods. The data points are average of two independent experiments, each comprising three biological repeats. Each biological repeat is represented by the average of three technical repeats. Data are shown as average at each time point. The standard error of mean (SEM) is not shown as there was very little variation.

A

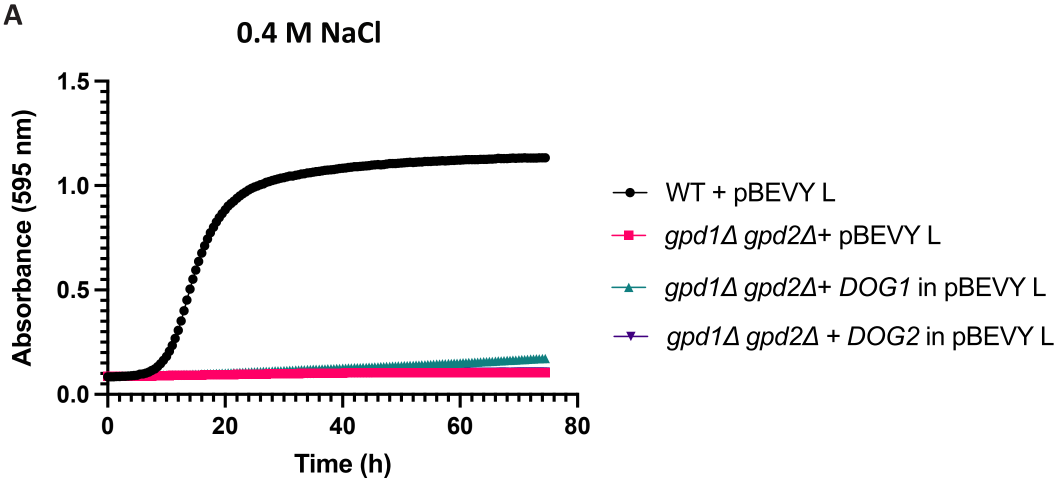

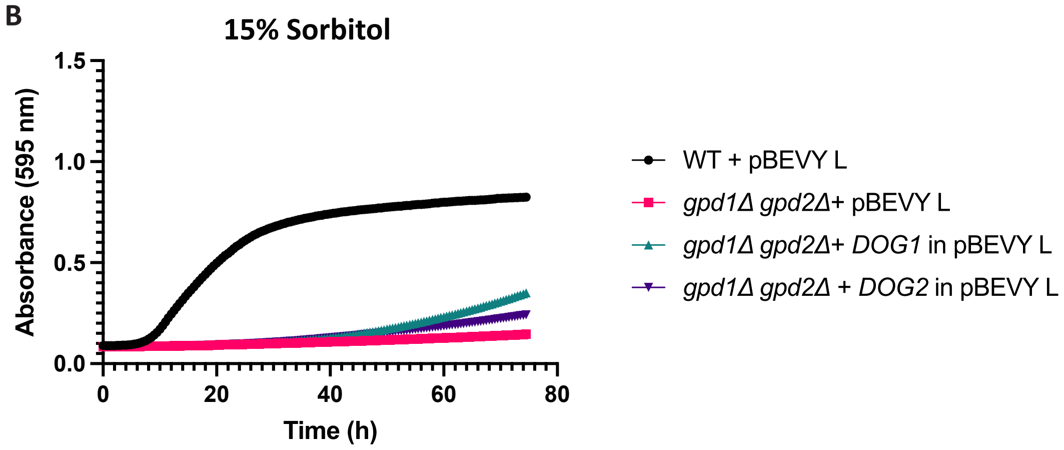

Supplementary FIG S3. *gpp1Δ gpp2Δ dog1Δ dog2Δ* quadruple mutant and *gpp1Δ gpp2Δ* double mutant behaves similarly when grown on salt stress. The liquid growth assays were done in medium containing 0.4 M NaCl for 72h as described in materials and methods. The data points are average of one experiment, each comprising three biological repeats. Each biological repeat is represented by the average of three technical repeats. Data are shown as average at each time point. The standard error of mean (SEM) is not shown as there was very little variation.

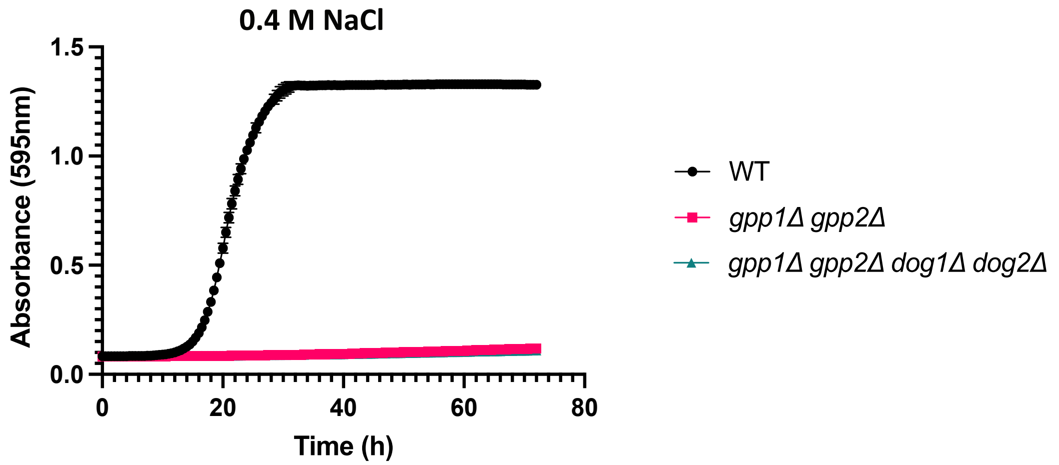

Supplementary FIG S4. Quantification of phosphate liberated by the Dog proteins. The dephosphorylating activity of purified Dog1 (panel A) and Dog2 (panel B) proteins at a concentration of 0.01 mg/ml on 1mM of 2-DG6P, DHAP and Gly-3P is depicted as the concentration of free phosphate liberated after the reaction in the presence of Mg2+ as a co-factor. Data are shown as averages and standard error of the mean (SEM).

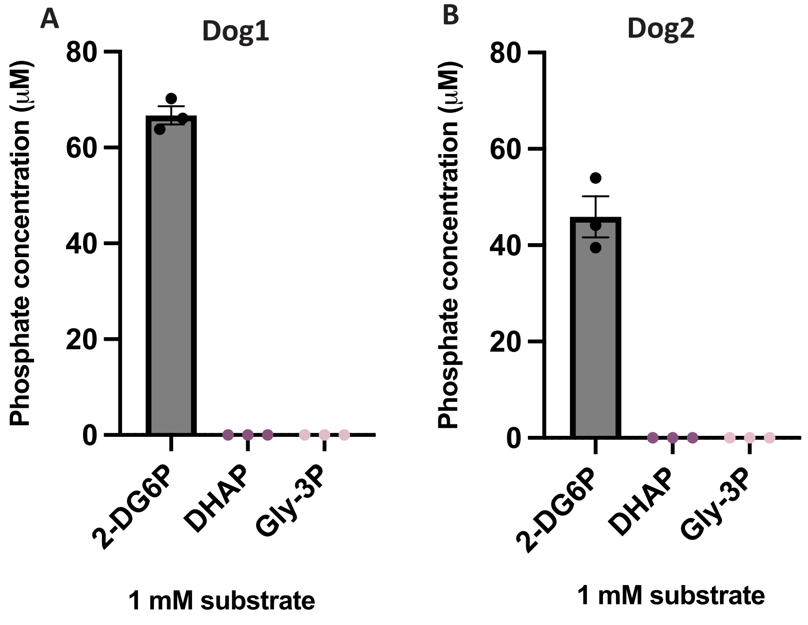

Supplementary FIG S5. Overexpression of the *DOG* genes do not improve the resilience of the WT towards ionic stress. The liquid growth assays were done in medium containing 0.8 M, 1 M, and 1.5 M NaCl for 72h as described in materials and methods. The data points are average of one experiment, each comprising three biological repeats. Each biological repeat is represented by the average of three technical repeats. Data are shown as average at each time point. The standard error of mean (SEM) is not shown as there was very little variation.

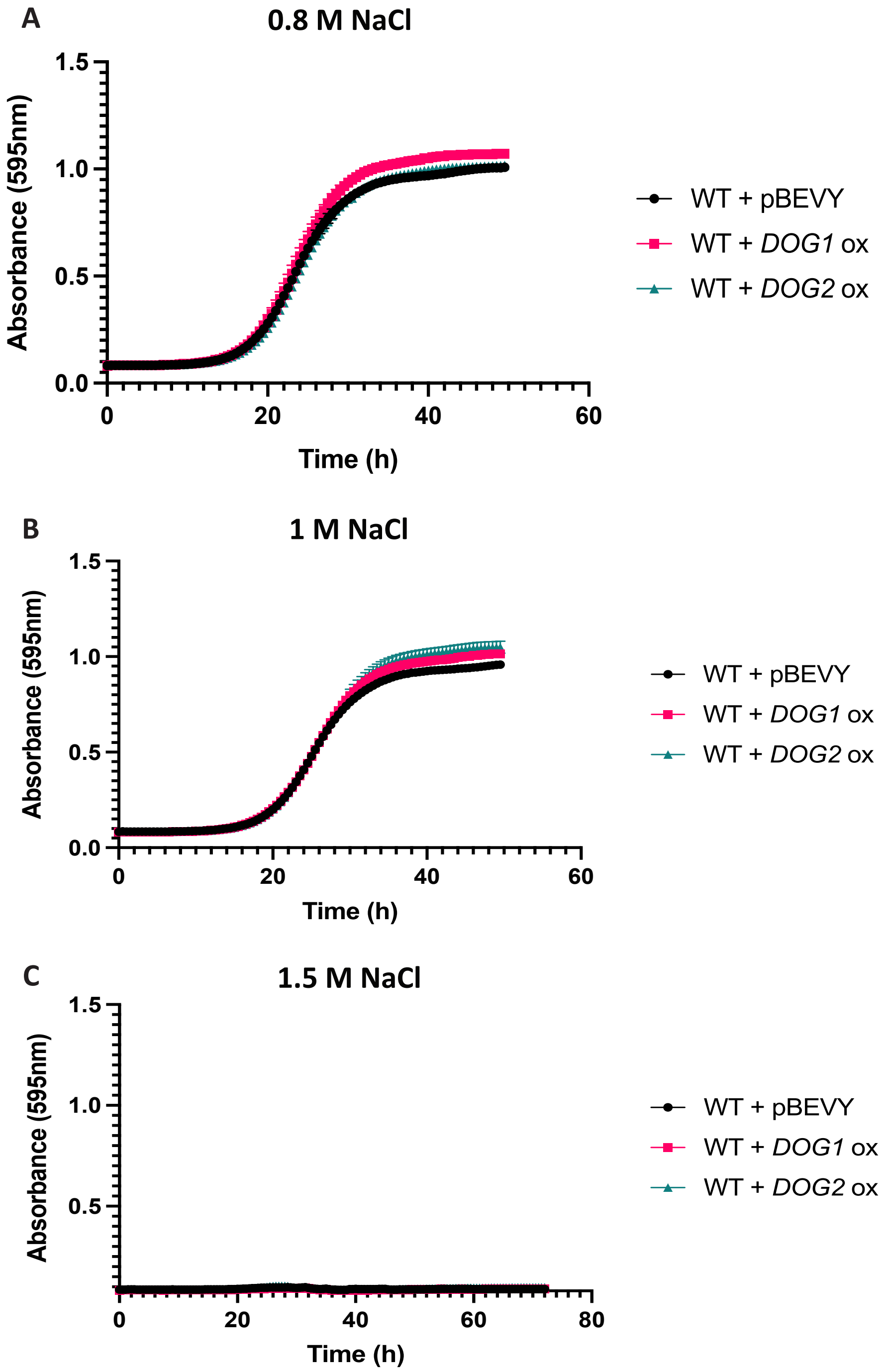

Supplementary FIG S6. Overexpression of the *DOG* genes do not suppress the growth sensitive phenotype of the *tps2Δ* mutant at 37°C. The liquid growth assay was done in CSM – leucine medium supplemented with 100 mg/L adenine and 2% glucose for 72h as described in materials and methods. The data points are average of one experiment, each comprising three biological repeats. Each biological repeat is represented by the average of three technical repeats. Data are shown as average at each time point with the standard error of mean (SEM).

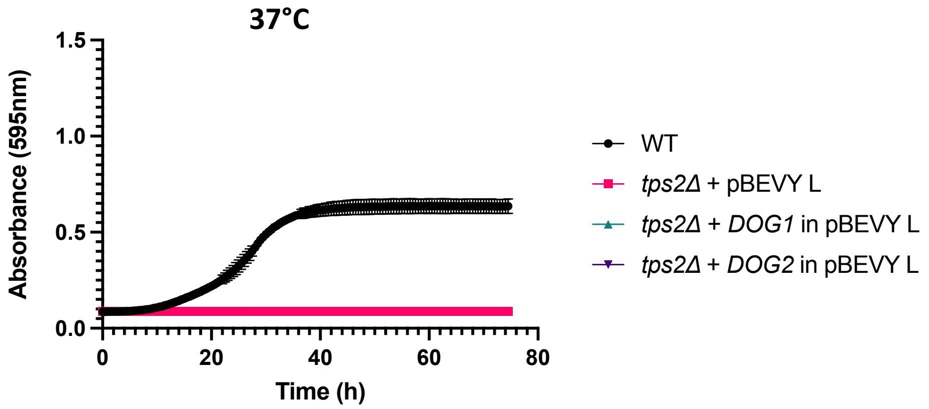

Supplement: Supplemental material — Fig. S1 to S6. [file spectrum.00136-24-s0001.pdf]
